# Supplementary material for: Modulate stress distribution with bio-inspired irregular architected materials towards optimal tissue support
Source: Nat Commun. 2024 May 21;15:4072. doi: 10.1038/s41467-024-47831-2 (PMC11109255; doi:10.1038/s41467-024-47831-2)
Supplement: Supplementary file 1 — Supplementary Information [file 41467_2024_47831_MOESM1_ESM.pdf]

Supplementary Information
Modulate stress distribution with bio-inspired irregular architected materials towards optimal tissue support

Yingqi Jia[a], Ke Liu[*,b], Xiaojia Shelly Zhang[*,a,c,d]

*Corresponding authors: Ke Liu (liuke@pku.edu.cn), Xiaojia Shelly Zhang (zhangxs@illinois.edu)

[a]Department of Civil and Environmental Engineering, University of Illinois Urbana-Champaign, Urbana, IL 61801, USA
[b]Department of Advanced Manufacturing and Robotics, Peking University, Beijing 100871, China
[c]Department of Mechanical Science and Engineering, University of Illinois Urbana-Champaign, Urbana, IL 61801, USA
[d]National Center for Supercomputing Applications, USA

1. Material database creation

1.1. Basic procedures

A material database stores the discrete relationships between the frequency combinations and the homogenized elastic moduli of disordered microstructures. This material database will be used to train a machine learning model and to predict mechanical responses. Below we present the detailed procedures for creating the material database (Supplementary Figure 1).

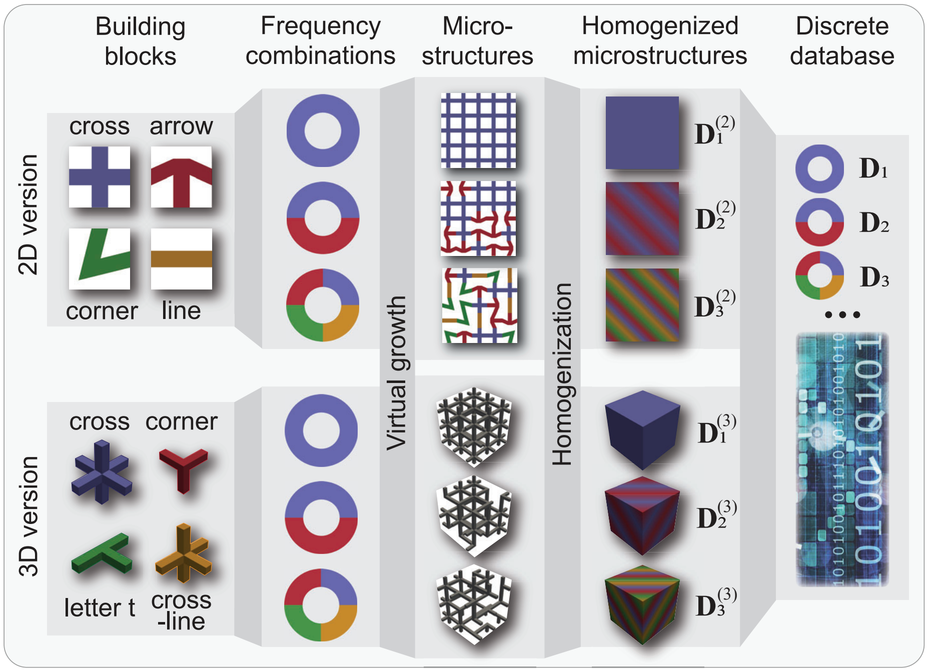

Supplementary Figure 1: **Material database creation in two- and three-dimensional cases.** This creation process involves specifying basic building blocks, sampling frequency combinations, generating disordered microstructures, and evaluating microstructural homogenized elastic moduli.

1.1.1. Prescribing basic building blocks

Basic building blocks are fundamental ingredients for creating disordered microstructures, and these microstructures further compose the irregular architected materials. To ensure the diversity and seamlessness of the generated materials, we require that the basic building blocks are representative and can be connected via rotation. Specifically, we prescribe four types of basic building blocks, namely cross, arrow, corner, and line shapes, in the two-dimensional (2D) case and four types of basic building blocks, namely cross, corner,

t, and cross-line shapes, in the three-dimensional (3D) case. These specified building blocks exhibit diverse geometries with each block capable of connecting only in specific directions to others, achieving a large material property space (see Supplementary Figure 3 that will be introduced later).

1.1.2. Sampling frequency combinations of microstructures

We denote one frequency combination of a disordered microstructure by $\{\bar{\xi}_1, \bar{\xi}_2, \bar{\xi}_3, \bar{\xi}_4\}$ satisfying $\sum_{i=1}^{4} \bar{\xi}_i = 1$, where $\bar{\xi}_1$–$\bar{\xi}_4$ are the frequencies of the four basic building blocks in the microstructure, respectively. To create a representative material database, we uniformly sample 200 microstructural frequency combinations on the hyperplane $\sum_{i=1}^{4} \bar{\xi}_i = 1$ for both 2D and 3D cases following the procedure in the Supporting Information of [1].

1.1.3. Generating disordered microstructures

Based on the prescribed basic building blocks and sampled frequency combinations, we use the original virtual growth scheme [1] to create 100 specimens of disordered microstructures for each frequency combination, resulting in 20,000 square specimens in 2D and 20,000 cubic specimens in 3D. Each 2D specimen contains 40×40 building blocks, and each 3D specimen contains $10 \times 10 \times 10$ building blocks.

1.1.4. Evaluating microstructural homogenized elastic modulus

The microstructure generated by the virtual growth scheme is irregular and should be homogenized to evaluate its elastic modulus. We use a numerical homogenization approach [2] (see also Section 1.2 for details) to evaluate the microstructural homogenized elastic modulus — a 3×3 matrix in 2D ($\mathbf{D}^{(2)}$) and a 6×6 matrix in 3D ($\mathbf{D}^{(3)}$), expressed in matrix notation [3]. We then compute the average elastic modulus for every 100 specimens corresponding to the same frequency combination. Finally, we obtain 200 pairs of frequency combination–elastic modulus relationships for both 2D and 3D cases.

1.2. Numerical homogenization of disordered microstructures

During the material database creation, we need to evaluate the homogenized elastic modulus ($\mathbf{D}$) of disordered microstructures. We achieve this goal by leveraging a numerical homogenization approach in [2]. This approach assumes that the microstructure under consideration is a unit cell of an infinitely large periodic structure, and we can compute the homogenized elastic modulus by imposing periodic boundary conditions on the unit cell (disordered microstructure herein). For the reader's convenience, we outline the key steps of this approach as follows.

We begin by specifying the periodic vectors, $\mathbf{a}_1$, $\mathbf{a}_2$, and $\mathbf{a}_3$, of the irregular microstructure. Next, we discretize the microstructure into finite elements and classify the degrees of freedom (DOFs) as independent or dependent. We then find the topological matrices, $\mathbf{B}_0$ and $\mathbf{B}_a$, such that

$$\mathbf{d} = \mathbf{B}_0 \mathbf{d}_0 + \mathbf{B}_a \Delta\mathbf{a} \tag{1}$$

where $\mathbf{d}$ and $\mathbf{d}_0$ are the displacement vectors of all DOFs and independent DOFs, respectively. The variable $\Delta\mathbf{a} = [\Delta\mathbf{a}_1^\top, \Delta\mathbf{a}_2^\top, \Delta\mathbf{a}_3^\top]^\top$ contains the changes in periodic vectors due to deformation, namely $\Delta\mathbf{a}_1$, $\Delta\mathbf{a}_2$, and $\Delta\mathbf{a}_3$. Note that Eq. (1) essentially represents the periodic boundary condition, and it implies a separation of lengths scales between the unit cell and the material piece.

Based on the finite element analysis (FEA), we evaluate the global stiffness matrix of the discretized microstructure as $\mathbf{K}_{\mathtt{uc}}$. In this study, we use first-order quadrilateral elements in 2D and frame elements in 3D to improve computational efficiency. By applying the periodic boundary conditions, we can establish the periodic equilibrium equation as

$$\mathbf{B}_0^\top \mathbf{K}_{\mathtt{uc}} \mathbf{d} = \mathbf{0}. \tag{2}$$

Substituting (1) into (2) yields

$$\mathbf{B}_0^\top \mathbf{K}_{\mathtt{uc}} (\mathbf{B}_0 \mathbf{d}_0 + \mathbf{B}_a \Delta\mathbf{a}) = \mathbf{0} \Rightarrow \mathbf{B}_0^\top \mathbf{K}_{\mathtt{uc}} \mathbf{B}_0 \mathbf{d}_0 = -\mathbf{B}_0^\top \mathbf{K}_{\mathtt{uc}} \mathbf{B}_a \Delta\mathbf{a},$$

and we obtain the displacement vector of independent DOFs as

$$\mathbf{d}_0 = -(\mathbf{B}_0^\top \mathbf{K}_{\mathtt{uc}} \mathbf{B}_0)^+ \mathbf{B}_0^\top \mathbf{K}_{\mathtt{uc}} \mathbf{B}_a \Delta\mathbf{a} := \mathbf{D}_0 \Delta\mathbf{a}. \tag{3}$$

Substituting (3) into (1) gives the displacement vector of all DOFs as

$$\mathbf{d} = (\mathbf{B}_0 \mathbf{D}_0 + \mathbf{B}_a)\Delta\mathbf{a} := \mathbf{D}_a \Delta\mathbf{a}.$$

We then evaluate the stored energy density as

$$W = \frac{1}{2V}\mathbf{d}^\top \mathbf{K}_{\mathtt{uc}} \mathbf{d} = \frac{1}{2V}\Delta\mathbf{a}^\top \mathbf{D}_a^\top \mathbf{K}_{\mathtt{uc}} \mathbf{D}_a \Delta\mathbf{a} := \frac{1}{2V}\Delta\mathbf{a}^\top \mathbf{K}_{\Delta\mathbf{a}} \Delta\mathbf{a}$$

where $V = \det([\mathbf{a}_1, \mathbf{a}_2, \mathbf{a}_3])$ is the microstructural volume.

We apply a dummy macroscopic strain field of $\boldsymbol{\varepsilon}_{M,3\times 3}$ on the microstructure. According to the definition of the infinitesimal strain, we have $\Delta\mathbf{a}_i = \boldsymbol{\varepsilon}_{M,3\times 3} \cdot \mathbf{a}_i$ for $i = 1, 2, 3$. We then can find a matrix, $\mathbf{B}_\varepsilon$, such that $\Delta\mathbf{a} = \mathbf{B}_\varepsilon \boldsymbol{\varepsilon}_{M,6\times 1}^V$ where $\boldsymbol{\varepsilon}_{M,6\times 1}^V$ is the Voight notation of $\boldsymbol{\varepsilon}_{M,3\times 3}$. Finally, we can rewrite the stored energy density as

$$W = \frac{1}{2V}(\boldsymbol{\varepsilon}_M^V)^\top \mathbf{B}_\varepsilon^\top \mathbf{K}_{\Delta\mathbf{a}} \mathbf{B}_\varepsilon \boldsymbol{\varepsilon}_M^V.$$

Based on continuum mechanics [3], the homogenized elastic modulus (in matrix notation) is given as

$$\mathbf{D} = \frac{1}{V}\mathbf{B}_\varepsilon^\top \mathbf{K}_{\Delta\mathbf{a}} \mathbf{B}_\varepsilon,$$

and we recall $\mathbf{K}_{\Delta\mathbf{a}} = \mathbf{D}_a^\top \mathbf{K}_{\mathtt{uc}} \mathbf{D}_a$, $\mathbf{D}_a = \mathbf{B}_0 \mathbf{D}_0 + \mathbf{B}_a$, $\mathbf{D}_0 = -(\mathbf{B}_0^\top \mathbf{K}_{\mathtt{uc}} \mathbf{B}_0)^+ \mathbf{B}_0^\top \mathbf{K}_{\mathtt{uc}} \mathbf{B}_a$, where $\mathbf{B}_0$, $\mathbf{B}_a$, $\mathbf{B}_\varepsilon$, $\mathbf{K}_{\mathtt{uc}}$, and V are ready to compute for a given microstructure.

1.3. Material property distribution

Considering the stochastic nature of the virtual growth algorithm, the material properties in the generated database exhibit randomness. In this subsection, we study the material property distribution of the created specimens. Supplementary Figure 2 displays the distribution of the D_{11} component of the homogenized elastic modulus ($\mathbf{D}$) for 100 specimens corresponding to randomly selected 9 frequency combinations. In Supplementary Figure 2, the x-axis represents the material property (D_{11} in this case). The left y-axis denotes the statistical frequency (count), while the right y-axis represents the probability density function. The inset in each panel presents the associated frequency combination of building blocks. Notably, the material properties of the generated specimens generally exhibit unimodality and near-zero skewness. Therefore, we assume that the material properties follow a normal distribution with their mean values determined by the frequency combinations of building blocks.

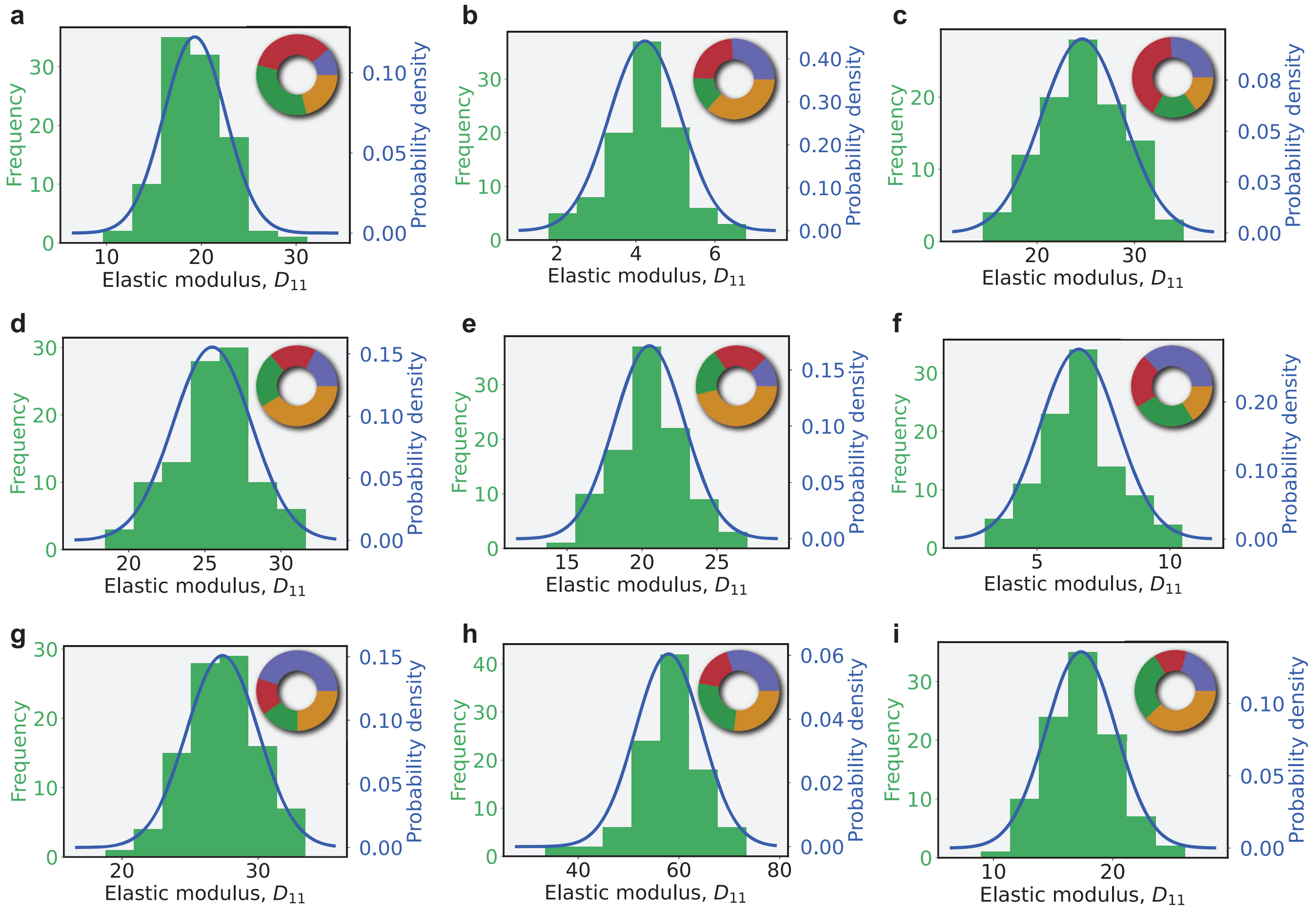

Supplementary Figure 2: **Material property distribution of the created specimens.** The x-axis is the material property (D_{11} component of the $\mathbf{D}$ matrix here), the left y-axis is the statistical frequency, and right y-axis is the probability density function. The inset color wheels represent the frequency combinations of building blocks.

1.4. Achieved material property space

In this subsection, we investigate the attained material property space based on the discrete material database in Supplementary Figure 1. Following the convention in [1], we begin by defining the average Young's modulus and Poisson ratio to quantify the homogenized anisotropic material properties of the microstructures in the database. Specifically, we define the average Young's modulus as

$$E^{\mathsf{ave}} = \begin{cases} \frac{1}{2}\left(\frac{1}{\mathbb{S}_{1111}} + \frac{1}{\mathbb{S}_{2222}}\right) & \text{in 2D,} \\ \frac{1}{3}\left(\frac{1}{\mathbb{S}_{1111}} + \frac{1}{\mathbb{S}_{2222}} + \frac{1}{\mathbb{S}_{3333}}\right) & \text{in 3D,} \end{cases}$$

and the average Poisson's ratio as

$$\nu^{\mathsf{ave}} = \begin{cases} -\frac{1}{2}\left(\frac{\mathbb{S}_{2211}}{\mathbb{S}_{1111}} + \frac{\mathbb{S}_{1122}}{\mathbb{S}_{2222}}\right) & \text{in 2D,} \\ -\frac{1}{6}\left(\frac{\mathbb{S}_{2211}}{\mathbb{S}_{1111}} + \frac{\mathbb{S}_{1122}}{\mathbb{S}_{2222}} + \frac{\mathbb{S}_{3322}}{\mathbb{S}_{2222}} + \frac{\mathbb{S}_{2233}}{\mathbb{S}_{3333}} + \frac{\mathbb{S}_{3311}}{\mathbb{S}_{1111}} + \frac{\mathbb{S}_{1133}}{\mathbb{S}_{3333}}\right) & \text{in 3D,} \end{cases}$$

where $\mathbb{S}$ represents the fourth-order homogenized compliance tensor that can be derived from the matrix $\mathbf{D}$.

With the above definitions, we plot the material property space for both 2D and 3D scenarios in Supplementary Figure 3. Here, the x-axis is the relative average Young's modulus defined as $E^{\mathsf{ave}}/E^{\mathtt{solid}}$, and

E^{solid} is the Young's modulus of the solid material; the y-axis is the average Poisson's ratio, ν^{ave}. In Supplementary Figure 3, the color wheels signify the frequency combinations present in the material database in Supplementary Figure 1, and their positions correspond to $E^{\text{ave}}/E^{\text{solid}}$–$\nu^{\text{ave}}$ pairs averaged over the specimens of the same frequency combination. The shaded area in Supplementary Figure 3 indicates the actual material properties achieved by all the specimens. The insets in both Supplementary Figures 3a and b illustrate specimens generated based on their frequency combinations, respectively. According to Supplementary Supplementary Figure 3, the 2D material property space spans $E^{\text{ave}}/E^{\text{solid}}$ values in [0.001, 0.111] and ν^{ave} values in [-0.309, 0.365]; the 3D material property space spans the counterparts in [0.003, 0.036] and [0.008, 0.144], respectively. We remark again that the broad spectrum of material properties results from the selection of the representative building blocks.

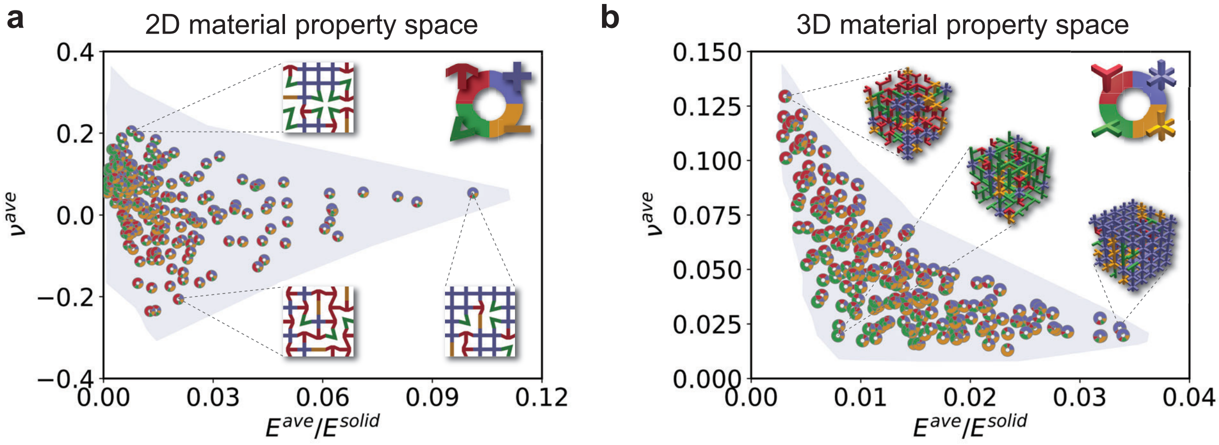

Supplementary Figure 3: **Material property space in 2D and 3D cases.** The x-axis is the relative average Young's modulus ($E^{\text{ave}}/E^{\text{solid}}$), and the y-axis is the average Poisson's ratio (ν^{ave}). The color wheels represent the frequency combinations in the material database, and their positions represent the corresponding $E^{\text{ave}}/E^{\text{solid}}$–$\nu^{\text{ave}}$ pairs averaged over specimens of the same frequency combination, respectively. The shaded regions indicate the actual material properties achieved by all the specimens, and the insets illustrate the generated microstructures.

2. Machine learning model for predicting microstructural elastic modulus

The above material database stores discrete relationships between the frequency combinations and the homogenized elastic modulus of disordered microstructures. However, we need continuous and differentiable relationships for the subsequent topology optimization. To capture such relationships, we train a machine learning model — a fully connected neural network — and use it to predict homogenized elastic modulus based on the input frequency combination. The architecture of the neural network as well as the training and testing processes are outlined below.

2.1. Architecture of the neural network

The architecture of the neural network is shown in Supplementary Figure 4. This neural network comprises one input layer with $N_b = 4$ nodes corresponding to the 4 entries of the input frequency combination. It also has two hidden layers with m and p nodes, respectively, and one output layer with q nodes corresponding to the maximum number of independent entries of homogenized elastic modulus ($q = 6$ in 2D and $q = 21$ in 3D). We employ the rectified linear activation unit, $\text{ReLU}(\cdot) = \max\{\cdot, 0\}$, as the nonlinear activation function. The mathematical expressions of the architecture in Supplementary Figure 4 are given as

$$\begin{cases} \boldsymbol{\zeta}_1^{m\times 1} = \mathbf{W}_1^{m\times N_b}\boldsymbol{\xi}^{N_b\times 1} + \mathbf{b}_1^{m\times 1} \\ \mathbf{h}_1^{m\times 1} = \text{ReLU}(\boldsymbol{\zeta}_1^{m\times 1}) \\ \boldsymbol{\zeta}_2^{p\times 1} = \mathbf{W}_2^{p\times m}\mathbf{h}_1^{m\times 1} + \mathbf{b}_2^{p\times 1} \\ \mathbf{h}_2^{p\times 1} = \text{ReLU}(\boldsymbol{\zeta}_2^{p\times 1}) \\ \mathbf{D}_{\text{vec}}^{q\times 1} = \mathbf{W}_3^{q\times p}\mathbf{h}_2^{p\times 1} + \mathbf{b}_3^{q\times 1} \end{cases}$$

where $\boldsymbol{\xi} = [\xi_1, \xi_2, ..., \xi_{N_b}]^\top$ is the input frequency combination. Variables, $\boldsymbol{\zeta}_1$, $\boldsymbol{\zeta}_2$, $\mathbf{h}_1 = [h_{1,1}, h_{1,2}, ..., h_{1,m}]^\top$, and $\mathbf{h}_2 = [h_{2,1}, h_{2,2}, ..., h_{2,p}]^\top$, are intermediate vectors used in the forward pass of the neural network. Weighting matrices, $\mathbf{W}_1$, $\mathbf{W}_2$, and $\mathbf{W}_3$, and bias vectors, $\mathbf{b}_1$, $\mathbf{b}_2$, and $\mathbf{b}_3$, are parameters to be optimized such that the prediction error of microstructural elastic modulus, $\mathbf{D}_{\texttt{vec}}$ (the vector form of $\mathbf{D}$), is minimized. Superscripts indicate the matrix and vector dimensions for the reader's convenience.

2.2. Training and testing the neural network

To optimize the weighting matrices ($\mathbf{W}_1$, $\mathbf{W}_2$, and $\mathbf{W}_3$) and bias vectors ($\mathbf{b}_1$, $\mathbf{b}_2$, and $\mathbf{b}_3$) of the neural network, we use the mean squared error (MSE) of the microstructural elastic modulus as the loss function ($\mathcal{L}$) and minimize it using an Adam optimizer [4]. To configure this optimizer, we set the coefficients used for computing running averages of gradient and its square as $\beta = (0.900, 0.999)$ and add a term $\varepsilon = 10^{-8}$ to the denominator to improve numerical stability. Before training the neural network, we also need to determine its optimal architecture for a faster convergence speed and a smaller value of the loss function, $\mathcal{L}$. To achieve this, we randomly test 100 sets of architecture parameters, including the learning rate of the Adam optimizer, $l \in \{10^{-5.0}, 10^{-4.5}, 10^{-4.0}, 10^{-3.5}, 10^{-3.0}, 10^{-2.5}, 10^{-2.0}\}$, and the numbers of nodes in the two hidden layers, m and $p \in \{4, 8, 16, 32, 64, 128, 256, 512\}$. We test each set of architecture parameters, (l, m, p), by running the neural network for 200 epochs. We finally select the architecture parameters as $l = 10^{-2}$, $m = 512$, and $p = 256$, giving the minimum loss, $\mathcal{L}$, as shown in Supplementary Figure 5a.

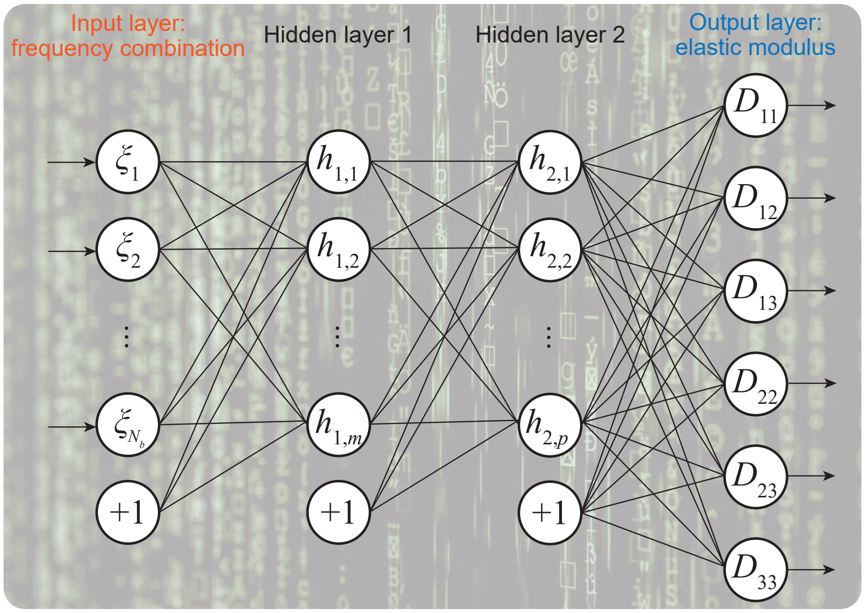

Supplementary Figure 4: **Architecture of a fully connected neural network for predicting homogenized elastic modulus.** The architecture contains one input layer with N_b nodes corresponding to the N_b entries of the input frequency combination. It also contains two hidden layers with m and p nodes, respectively, and one output layer with 6 nodes in 2D and 21 nodes in 3D corresponding to the maximum number of independent entries of homogenized elastic modulus.

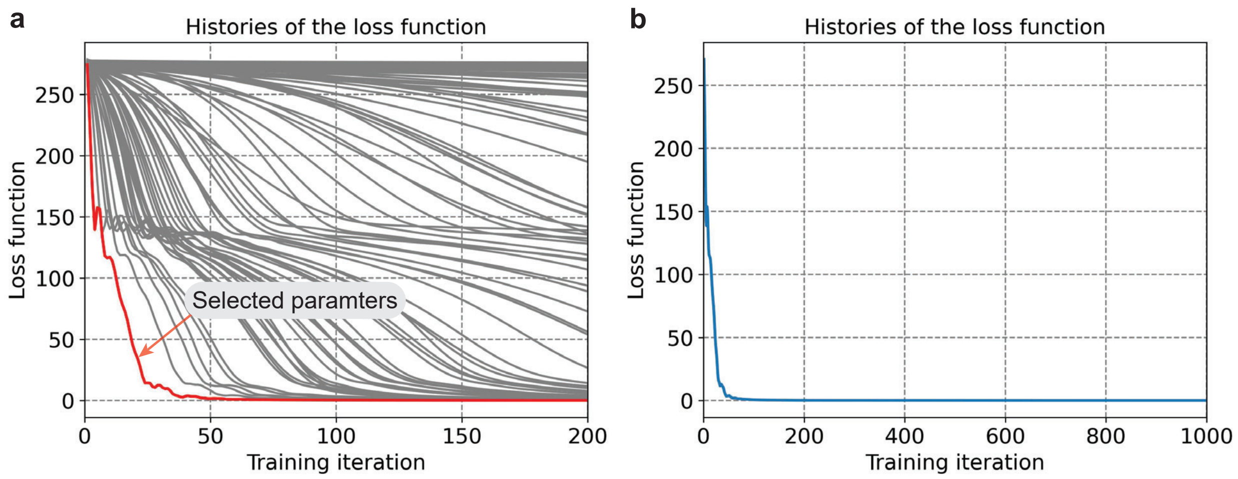

Supplementary Figure 5: **Determining the optimal architecture parameters and formally training the neural network.** **a**, Using a stochastic search to determine the optimal architecture parameters of the neural network. This figure shows the histories of the loss functions of 100 sets of randomly generated architecture parameters, including the learning rate of the optimizer and the numbers of nodes of the two hidden layers. **b**, The complete history of the loss function during the formal training of weighting matrices and bias vectors.

Based on the selected architecture parameters of the neural network, we now formally train its weighting matrices ($\mathbf{W}_1$, $\mathbf{W}_2$, and $\mathbf{W}_3$) and bias vectors ($\mathbf{b}_1$, $\mathbf{b}_2$, and $\mathbf{b}_3$). Specifically, we randomly set 160 frequency combination–elastic modulus pairs in the previous material database as training data and the remaining 40 pairs as testing data. We train the neural network for 1000 epochs using an adaptive learning rate: 10^{-2} for epochs 1–200, 10^{-3} for epochs 201–500, and 10^{-4} for epochs 501-1000. We implement and train this neural network with an in-house Python code [5] and the PyTorch package [6]. After the training, the loss function of the neural network ($\mathcal{L}$) converges to a sufficiently small value (Supplementary Figure 5b). Based on the testing data, the neural network performs well in predicting the microstructural homogenized elastic modulus with small MSEs of 0.134 MPa in 2D and 0.296 MPa in 3D, compared to their corresponding mean values of 2.687 MPa and 7.344 MPa, respectively.

3. Macroscopic topology optimization for stress modulation

Based on the continuous relationship between the microstructural frequency combination and elastic modulus, we can establish a macroscopic topology optimization formulation for stress modulation. This formulation consists of design space parameterization, elasticity interpolation, and static equilibrium equations, which are introduced below. After that, we introduce the complete formulation and show the detailed problem setups for stress modulation.

3.1. Design space parameterization

We need to parameterize the design space to optimize the microstructural layout and frequency combination. Specifically, we parameterize the microstructural layout with a density field, $\rho(\mathbf{X}) \in [0, 1]$, and parameterize the microstructural frequency combination with frequency fields, $\xi_i(\mathbf{X}) \in [\varepsilon, 1-\varepsilon]$ for $i = 1, 2, ..., N_b$, where N_b represents the number of types of basic building blocks, and $\varepsilon \in [0, 0.5)$ is a prescribed constant. The variable $\mathbf{X}$ is the position vector of a material point in the design domain, Ω.

To reduce the mesh dependency and eliminate the checkerboard pattern of the density field, $\rho(\mathbf{X})$, during topology optimization, we apply a density filter [7] and derive the filtered density field as

$$\widetilde{\rho}(\mathbf{X}) = \frac{\displaystyle\int_{\Omega} w(\mathbf{X}, \mathbf{X}')\rho(\mathbf{X}')\, \mathrm{d}\mathbf{X}'}{\displaystyle\int_{\Omega} w(\mathbf{X}, \mathbf{X}')\, \mathrm{d}\mathbf{X}'},$$

where the weighting factor, $w(\mathbf{X}, \mathbf{X}') = \max\{0, R - |\mathbf{X} - \mathbf{X}'|\}$, is determined by the filter radius (R) and the Euclidean distance ($|\mathbf{X} - \mathbf{X}'|$) between material points $\mathbf{X}$ and $\mathbf{X}'$. To ensure the 0–1 solution of the density field, we further apply a Heaviside projection [8] and derive the physical density field as

$$\overline{\rho}(\mathbf{X}) = \frac{\tanh(\beta\theta) + \tanh(\beta(\widetilde{\rho}(\mathbf{X}) - \theta))}{\tanh(\beta\theta) + \tanh(\beta(1 - \theta))},$$

where $\theta = 0.5$ is a threshold value and β is a sharpness parameter. The physical density $\overline{\rho}(\mathbf{X}) = 1$ represents the presence of disordered microstructures, and $\overline{\rho}(\mathbf{X}) = 0$ represents the voids.

As for the frequency fields, they need to satisfy the axiom of probability, $\sum_{i=1}^{N_b} \xi_i(\mathbf{X}) = 1$. To effectively impose this constraint, we propose a normalization projection scheme and obtain the physical frequency fields as

$$\overline{\xi}_i(\mathbf{X}) = \frac{\xi_i(\mathbf{X})}{\sum_{j=1}^{N_b} \xi_j(\mathbf{X})} \in [\frac{\varepsilon}{N_b - 1 + (2 - N_b)\varepsilon}, \frac{1 - \varepsilon}{1 + (N_b - 2)\varepsilon}] \quad \text{for } i = 1, 2, ..., N_b \text{ and } N_b \geq 2,$$

where $\overline{\xi}_i(\mathbf{X})$ represents the i-th component of the microstructural frequency combination.

3.2. Elasticity interpolation and static equilibrium equations

After parameterizing the design space with the physical density ($\overline{\rho}(\mathbf{X})$) and frequency ($\overline{\xi}_i(\mathbf{X})$ for $i = 1, 2, ..., N_b$) fields, we need to interpolate the microstructural elastic modulus ($\mathbb{C}(\mathbf{X})$, in a tensorial form) for these variables. Based on the modified solid isotropic material with penalization (SIMP) [9], the interpolated microstructural elastic modulus is given as

$$\mathbb{C} = [\varepsilon_\rho + (1 - \varepsilon_\rho)\overline{\rho}^{p_\rho}] \cdot \left[(1 - \alpha) \cdot \mathbb{C}_d(\overline{\xi}_1, \overline{\xi}_2, ..., \overline{\xi}_{N_b}) + \alpha \cdot \mathbb{C}_p\right],$$

where $\varepsilon_\rho = 10^{-6}$ is a small positive number preventing the singularity of the stiffness matrix in FEA. The parameter $p_\rho = 3$ penalizes the intermediate values of the physical density field, $\overline{\rho} \in (0, 1)$, and leads to a 0–1 solution of $\overline{\rho}$. The parameter α characterizes the designable region as $\alpha = 0$ and the passive region as $\alpha = 1$. The variables $\mathbb{C}_d$ and $\mathbb{C}_p$ are the corresponding elastic moduli, respectively, where $\mathbb{C}_d$ can be predicted by the trained machine learning model according to the inputs of $\overline{\xi}_1, \overline{\xi}_2, ..., \overline{\xi}_{N_b}$.

Based on the interpolated microstructural elastic modulus, we establish the static equilibrium equations as [3]

$$\begin{cases} \mathrm{Div}(\mathbb{C} : \mathbf{E}) + \overline{\mathbf{b}} = \mathbf{0}, & \mathbf{X} \in \Omega, \\ \mathbf{u} = \overline{\mathbf{u}}, & \mathbf{X} \in \partial\Omega_{\mathcal{D}}, \\ (\mathbb{C} : \mathbf{E}) \cdot \mathbf{N} = \overline{\mathbf{t}}, & \mathbf{X} \in \partial\Omega_{\mathcal{N}}, \end{cases} \tag{4}$$

where the variable $\mathbf{E} = (\nabla\mathbf{u} + \nabla\mathbf{u}^\top)/2$ is the infinitesimal strain tensor, and $\mathbf{u}$ is the displacement field. The variable $\overline{\mathbf{b}}$ is the applied body force, and $\overline{\mathbf{u}}$ is the displacement field prescribed on one part of the boundary, $\partial\Omega_{\mathcal{D}}$. The variable $\overline{\mathbf{t}}$ is the traction applied on the remaining part of the boundary, $\partial\Omega_{\mathcal{N}}$, with a unit outward normal vector, $\mathbf{N}$.

3.3. Macroscopic topology optimization formulation

Based on the design space parameterization and static equilibrium equations, we can employ topology optimization to modulate stress distribution. Topology optimization [10, 8] is an approach that determines the material layout that optimizes a given objective function while satisfying certain constraints. Leveraging topology optimization, researchers successfully design optimal structures and materials with complex programmable behaviors [11, 12, 13, 14]. In this work, we propose a macroscopic topology optimization

formulation for stress modulation, which is expressed as

$$
\begin{cases}
\underset{\rho,\ \xi_1,\ \xi_2,\ \dots,\ \xi_{N_b}}{\text{minimize}} & : \quad J = \sum_{l=1}^{N_l} w^{[l]} \left[\sum_{s=1}^{N_s} \int_\Omega \left(\theta^{[l,s]} \cdot \left| \frac{\sigma^{[l,s]} - \overline{\sigma}^{[l,s]}}{\overline{\sigma}^{[l,s]}} \right| \right)^p \mathrm{d}\mathbf{X} \right]^{\frac{1}{p}}, \\
\text{subjected to:} & \quad g = \frac{1}{|\Omega|} \int_\Omega \overline{\rho} \mathrm{d}\mathbf{X} - \overline{V} \leq 0, \\
 & \quad 0 \leq \rho \leq 1, \\
 & \quad \varepsilon \leq \xi_i \leq 1 - \varepsilon, \quad \text{for } i = 1, 2, ..., N_b, \\
\text{with:} & \quad \text{static equilibrium equations in (4)},
\end{cases}
$$

where J is the objective function representing the stress modulation error accounting for N_s stress measures and N_l load cases. The parameter $w^{[l]} \in [0,1]$ is the weighing factor of the l-th load case and satisfies $\sum_{l=1}^{N_l} w^{[l]} = 1$. The parameter $\theta^{[l,s]}(\mathbf{X})$ is the indicator of stress control regions of the s-th stress measure under the l-th load case, where $\theta^{[l,s]} = 1$ represents stress control regions, and $\theta^{[l,s]} = 0$ represents non-controlled regions. The variable $\sigma^{[l,s]}(\mathbf{X})$ is a stress measure, and $\overline{\sigma}^{[l,s]}(\mathbf{X})$ is the corresponding target value. The parameter p is used in the p-norm expression to approximate the maximum stress modulation error. The symbol g represents a volume constraint function, and the parameter $\overline{V}$ represents the allowable volume fraction of disordered microstructures.

To implement this proposed macroscopic topology optimization formulation, we utilize an in-house Python code [5] with the open-source FEniCSx package [15], which features parallel computing and automatic differentiation for the FEA and sensitivity analysis. We also use the method of moving asymptotes (MMA) [16] to update the design variables, ρ and ξ_i for $i = 1, 2, ..., N_b$.

3.4. Detailed problem setups and additional investigations

Based on the proposed macroscopic topology optimization framework, we move forward to optimize the microstructural layout and frequency combination to minimize the stress modulation error. We consider four representative scenarios and show the detailed problem setups and additional investigations as follows.

3.4.1. Manipulating mechanical stress distribution in varied geometric regions

The design domain is 180×90 mm^2, and we use a structured background mesh consisting of 30×15 rectangular elements for Cases 1 and 2 and use an unstructured background mesh with a similar element size for Case 3. We set the maximum optimization iteration as 150 and $p = 2$ in the objective function. In the main manuscript, we modulate the hydrostatic stress to a target value of $\overline{\sigma}^h = 0.28$ MPa under an applied displacement of $u = 1.5$ mm. The selection of this setup is solely for demonstration purposes, and the proposed computational framework is not confined to a specific stress measure, target value, or applied displacement. To illustrate the versatility of the framework, we take Case 2 as an example and explore stress modulation effects under varying setups. Supplementary Figure 6 displays the initial, optimized, and target stresses, with the insets illustrating the design setup. Specifically, in Supplementary Figure 6a, we modulate the shear stress (instead of hydrostatic stress) to two target values, $\overline{\tau} = 0.02$ MPa and $\overline{\tau} = 0.04$ MPa, respectively. In Supplementary Figure 6b, we explore two different target values for hydrostatic stress, $\overline{\sigma}^h = 0.18$ MPa and $\overline{\sigma}^h = 0.38$ MPa (as opposed to $\overline{\sigma}^h = 0.28$ MPa). Finally, in Supplementary Figure 6c, we consider two different applied displacements, $u = 0.20$ mm and $u = 0.25$ mm (instead of $u = 0.15$ mm). Supplementary Table 1 summarizes the design setups and the corresponding relative stress modulation errors. According to Supplementary Figure 6 and Supplementary Table 1, the stress distribution aligns with the target after optimization across diverse setups, indicating the robust generality of the proposed framework for stress modulation.

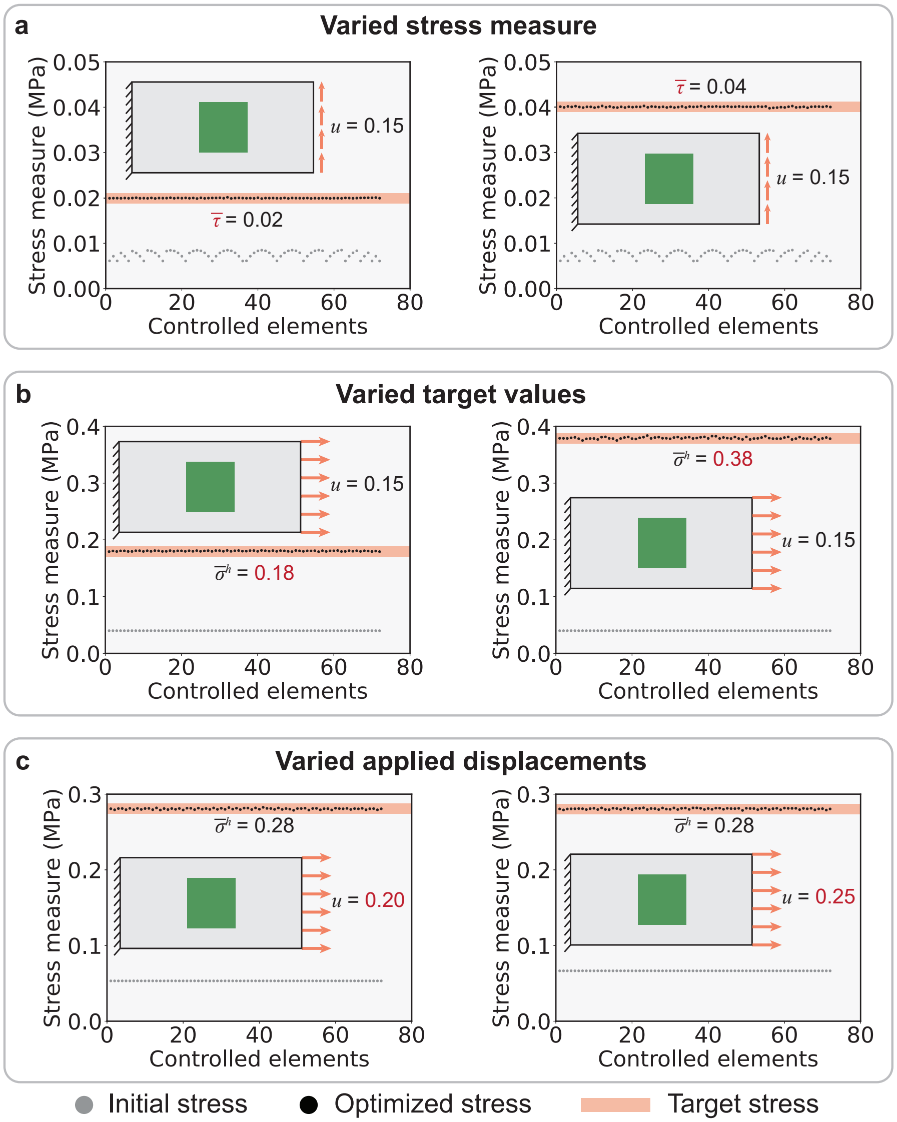

Supplementary Figure 6: **Investigation of stress modulation effects under varying setups.** **a**, The stress measure is changed from hydrostatic to shear stress. **b**, The target value is changed from 0.28 MPa to 0.18 and 0.38 MPa. **c**, The applied displacement is changed from 0.15 mm to 0.20 and 0.25 mm. The insets illustrate the updated design setups.

Supplementary Table 1: Summary of design setups and relative stress errors in Supplementary Figure 6

**Stress measure**	**Target value (MPa)**	**Applied displacement (mm)**	**Loading direction**	**Initial stress error**	**Optimized stress error**
Shear ($\overline{\tau}$)	0.02	0.15	Upward	532.0%	2.8%
Shear ($\overline{\tau}$)	0.04	0.15	Upward	690.0%	2.6%
Hydrostatic ($\overline{\sigma}^h$)	0.18	0.15	Rightward	660.0%	3.2%
Hydrostatic ($\overline{\sigma}^h$)	0.38	0.15	Rightward	759.0%	4.0%
Hydrostatic ($\overline{\sigma}^h$)	0.28	0.20	Rightward	687.0%	3.2%
Hydrostatic ($\overline{\sigma}^h$)	0.28	0.25	Rightward	647.0%	3.2%

3.4.2. Simultaneous mechanical stress modulation in multiple complex regions with distinct target values

The design domain is 350×100 mm^2, and we use a structured background mesh consisting of 875×250 rectangular elements. We set the maximum optimization iteration as 200 and $p = 2$ in the objective function.

In the main manuscript, we set the target stress values as 0.1, 0.3, and 0.5 MPa for distinct control regions. Here, we explore the stress modulation effects for other magnitudes of target values. In Supplementary Figure 7a, we consider 0.01, 0.03, and 0.05 MPa as the target values, and all the other setups remain unchanged. Likewise, we consider 0.2, 0.6, and 1.0 MPa as the target values in Supplementary Figure 7b. We observe that the actual stress distribution closely aligns with the target for both scenarios, demonstrating the robust stress modulation capability of the proposed framework.

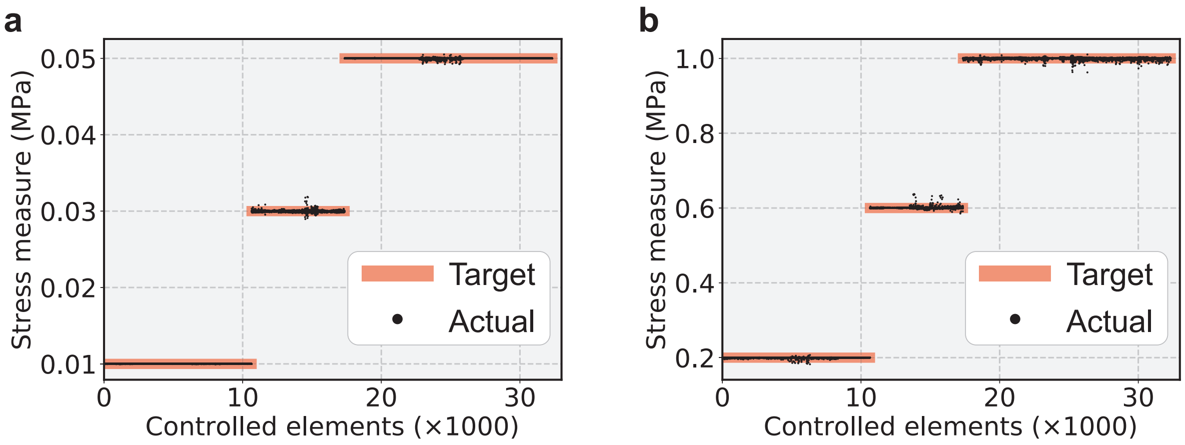

Supplementary Figure 7: **Stress modulation effects for different magnitudes of target values.** **a**, The target stress values are 0.01, 0.03, and 0.05 MPa. **b**, The target stress values are 0.2, 0.6, and 1.0 MPa.

3.4.3. Lightweight architected materials for multifunctional stress modulation

The design domain is 120×120 mm^2, and we use an unstructured background mesh with an element size of 1.5 mm. The filter radius of the physical density field is $R = 7.5$ mm, and the sharpness parameter is $\beta = 1$ initially and doubled every 40 optimization iteration starting from iteration 51 until $\beta = 256$. The allowable volume fraction of disordered microstructures is $\overline{V} = 0.7$. We also set the maximum optimization iteration as 400 and $p = 2$ in the objective function.

3.4.4. Potential application to orthopedic femur restoration

We use an unstructured background mesh consisting of 8292 tetrahedral elements. We set the maximum optimization iteration as 400 and $p = 16$ in the objective function. In Supplementary Figure 8, we present both the mesh discretization and the displacement field in the z direction after optimization. Upon close examination of the displacement field in the zoomed-in view, we observe that the relative displacement of the two femur fragments perpendicular to the fracture is approximately 0.3 mm. This value falls within the range of [0.2, 1.0] mm, suggesting the potential to facilitate femur restoration [17].

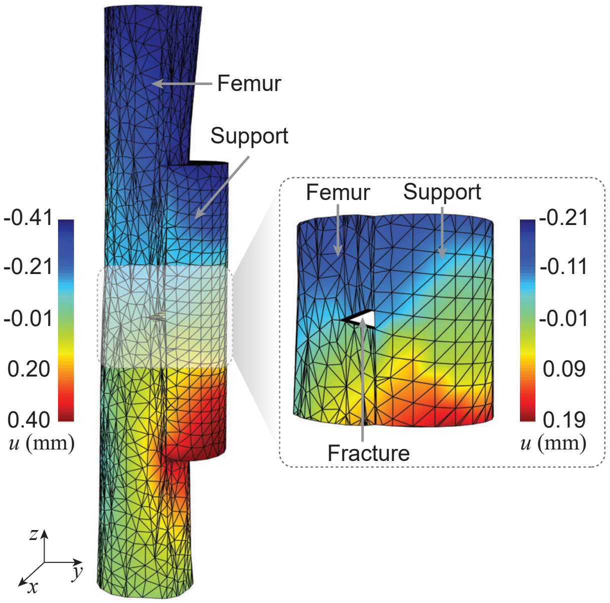

Supplementary Figure 8: **Finite element discretization and the optimized displacement field in z-direction of orthopedic femur restoration.** The model consists of the femur embedded with a pre-fracture and artificial support. Both components are discretized with tetrahedral elements due to complex geometries. In the visualization, color signifies the optimized displacement field in the z-direction, with the inset offering a zoom-in view of the displacement field around the fracture.

4. Bio-inspired virtual growth simulator

Based on the optimized physical density and frequency fields obtained from macroscopic topology optimization, we can utilize a bio-inspired virtual growth simulator — extended from the original virtual growth scheme [1] — to generate irregular architected materials with heterogeneous disordered microstructures. In this section, we introduce the basic procedures of this virtual growth simulator and the involved two-mesh-projection scheme.

4.1. Basic procedures of the virtual growth simulator

During the macroscopic topology optimization, we discretize a design domain with a (either structured or unstructured) background mesh and obtain the optimized density and frequency fields defined on this background mesh. Supplementary Figure 9a shows one representative background mesh consisting of four grids: one solid grid ($\overline{\rho} = 1$), one void grid ($\overline{\rho} = 0$), and two designable grids associated with an optimized frequency combination, $\{\overline{\xi}_1, \overline{\xi}_2, ..., \overline{\xi}_{N_b}\}$. To utilize these optimized fields for guiding the material generation, we propose a virtual growth simulator consisting of the following basic procedures: defining a structured foreground mesh fully covering the background mesh (Supplementary Figure 9b), projecting the optimized density and frequency fields onto the defined foreground mesh (Supplementary Figure 9c; see details in Section 4.2), growing disordered microstructures based on the projected fields, and cutting the foreground mesh to fit the geometry of the background mesh (Supplementary Figure 9d). Finally, we obtain seamless integration of irregular architected materials with optimized heterogeneous disordered microstructures (Supplementary Figure 9d).

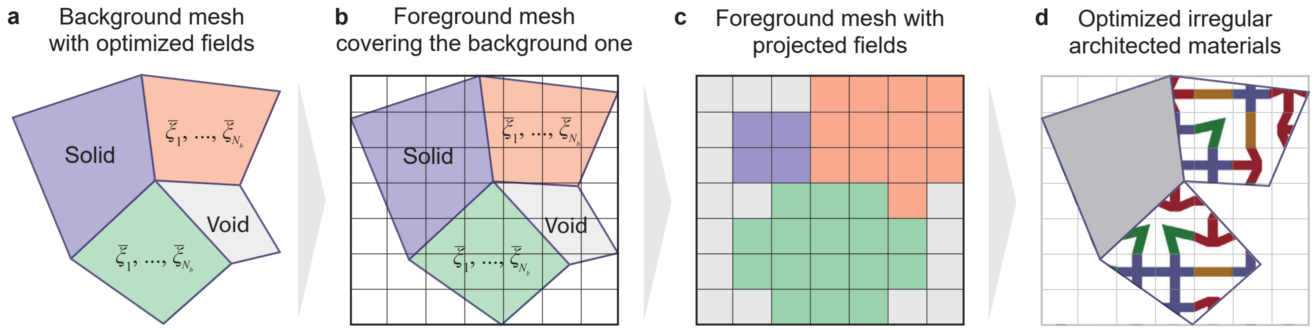

Supplementary Figure 9: **The proposed virtual growth simulator.** **a**, One representative unstructured background mesh consisting of four elements. **b**, A structured foreground mesh defined to cover the unstructured background mesh. **c**, The structured foreground mesh with projected density and frequency fields. **d**, Optimized irregular architected materials cut to match the geometries of the background mesh.

4.2. Two-mesh-projection scheme

As mentioned above, we need to project the optimized density and frequency fields onto a structured foreground mesh within the virtual growth simulator (Supplementary Figure 9b–c). This is because the original virtual growth scheme [1] is only applicable for a structured mesh with either square (in 2D) or cubic (in 3D) elements. To accomplish this goal, we propose a two-mesh-projection scheme to determine the corresponding source background grid for each foreground one. Specifically, we calculate the area (in 2D) or volume (in 3D) ratio between each pair of the intersected region (between background and foreground grids) and the foreground grid. The foreground grid then inherits the optimized density and frequency fields of the background grid associated with the highest area or volume ratio. In addition, a foreground grid is classified as solid if it is entirely within one or more solid background grids; a foreground grid is classified as void if it does not intersect with any designable background grids and is not classified as solid. These rules ensure that the obtained irregular architected materials are still fully connected after we cut them to match the geometries of the background mesh (Supplementary Figure 9d).

The pseudo-code of the proposed two-mesh-projection scheme is given in Algorithm 1. The proposed scheme involves computing the area ratios between polygons (in 2D) or volume ratios between polyhedra (in 3D) for each pair of the intersected region and the foreground grid. To accomplish this goal, we subdivide each background and foreground grid into many subgrids. We then calculate the central coordinates of each subgrid and use these coordinates to determine the nearest background subgrid for each foreground subgrid. By associating each foreground subgrid with the background grid containing the nearest background subgrid, we can approximate the area or volume ratios between the intersected region and the foreground grid. To effectively perform these complex geometric operations, we use an in-house Python code [5] with the PyVista [18], GeoPandas [19], and Shapely [20] packages. Notably, our implementation has no restrictions on the geometries of both the background and foreground meshes, enabling the projection of optimized fields from a structured/unstructured background mesh onto another structured/unstructured foreground mesh.

Algorithm 1: Two-mesh-projection scheme for projecting the optimized fields

Inputs: the number of foreground grids, $N^{\texttt{fore}}$; the number of background subgrids in one background grid, $N^{\texttt{back}}_{\texttt{sub}}$; the number of foreground subgrids in one foreground grid, $N^{\texttt{fore}}_{\texttt{sub}}$;
Find all solid and void background grids, $\mathcal{S}$ and $\mathcal{V}$, respectively;
Subdivide each background grid into $N^{\texttt{back}}_{\texttt{sub}}$ subgrids;
Subdivide each foreground grid into $N^{\texttt{fore}}_{\texttt{sub}}$ subgrids;
Initialize the foreground grid count, $n^{\texttt{fore}} = 1$;
while $n^{\texttt{fore}} \leq N^{\texttt{fore}}$ **do**
  Initialize a relation vector, $\mathbf{R} = \mathbf{0} \in \mathbb{R}^{N^{\texttt{fore}}_{\texttt{sub}}}$. Here $R_i = j$ represents that subgrid i of foreground grid $n^{\texttt{fore}}$ is within background grid j; subgrid i does not belong to any background grid if $R_i = 0$;
  Initialize the foreground subgrid count, $n^{\texttt{fore}}_{\texttt{sub}} = 1$;
  while $n^{\texttt{fore}}_{\texttt{sub}} \leq N^{\texttt{fore}}_{\texttt{sub}}$ **do**
    if foreground subgrid $n^{\texttt{fore}}_{\texttt{sub}}$ is within the background mesh **then**
      Find the nearest background subgrid and the associated background grid, k;
      $R_{n^{\texttt{fore}}_{\texttt{sub}}} \leftarrow k$;
    end
    $n^{\texttt{fore}}_{\texttt{sub}} \leftarrow n^{\texttt{fore}}_{\texttt{sub}} + 1$;
  end
  Let $\mathcal{U}$ denote unique entries in $\mathbf{R}$;
  if $\mathcal{U} \subseteq \mathcal{S}$ **then**
    Foreground grid $n^{\texttt{fore}}$ is solid;
  else if $\mathcal{U} \subseteq \mathcal{S} \cup \mathcal{V} \cup \{0\}$ **then**
    Foreground grid $n^{\texttt{fore}}$ is void;
  else
    Remove all entries in $\mathbf{R}$ if they are also in $\mathcal{S} \cup \mathcal{V} \cup \{0\}$;
    Foreground grid $n^{\texttt{fore}}$ inherits the optimized density and frequency fields of background grid $n = \text{Mode}(\mathbf{R})$;
  end
  $n^{\texttt{fore}} \leftarrow n^{\texttt{fore}} + 1$;
end

5. Stress convergence study

5.1. Average stress measures

To conduct a stress convergence study for irregular architected materials, we need to determine an appropriate measure for evaluating the average stress. We start from the stress tensor, $\boldsymbol{\sigma}(\mathbf{X})$, averaged over background grid e, which can be expressed as [21]

$$\langle \boldsymbol{\sigma} \rangle_e = \frac{1}{|\Omega_e|} \int_{\Omega_e} \boldsymbol{\sigma}(\mathbf{X}) \mathrm{d}\mathbf{X}$$

where $\langle \boldsymbol{\sigma} \rangle_e$ is the average stress tensor, and $\langle \cdot \rangle$ is the operator for computing the volume average. The variable Ω_e is the domain occupied by background grid e.

We now consider an arbitrary stress measure, $\sigma^s = f(\boldsymbol{\sigma})$, as a function of the stress tensor, $\boldsymbol{\sigma}$. The average stress measure can be computed as

$$\overline{\sigma}^s_e = f(\langle \boldsymbol{\sigma} \rangle_e),$$

and it holds

$$\overline{\sigma}^s_e = \langle f(\boldsymbol{\sigma}) \rangle_e = \langle \sigma^s \rangle_e$$

if the stress measure, σ^s, is a linear combination of the components (σ_{ij} for $i, j = 1, 2, 3$) of the stress tensor, $\boldsymbol{\sigma}$.

In this study, we mainly focus on the hydrostatic stress and the shear stress, and both of them are linear functions of the stress components. Therefore, the macroscopic average stress measure (Supplementary Figure 9a) is given as

$$\overline{\sigma}_e^{s,\texttt{macro}} = \langle \sigma^{s,\texttt{macro}} \rangle_e = \frac{1}{|\Omega_e|} \int_{\Omega_e} \sigma^{s,\texttt{macro}}(\mathbf{X}) \mathrm{d}\mathbf{X}, \tag{5}$$

and the microscopic average stress measure (Supplementary Figure 9d) is given as

$$\overline{\sigma}_e^{s,\texttt{micro}} = \langle \sigma^{s,\texttt{micro}} \rangle_e = \frac{1}{|\Omega_e|} \int_{\Omega_e} \sigma^{s,\texttt{micro}}(\mathbf{X}) \mathrm{d}\mathbf{X} \tag{6}$$

where $\sigma^{s,\texttt{macro}}$ and $\sigma^{s,\texttt{micro}}$ are macroscopic and microscopic stress measures of interest, respectively.

5.2. Convergence of the average stress

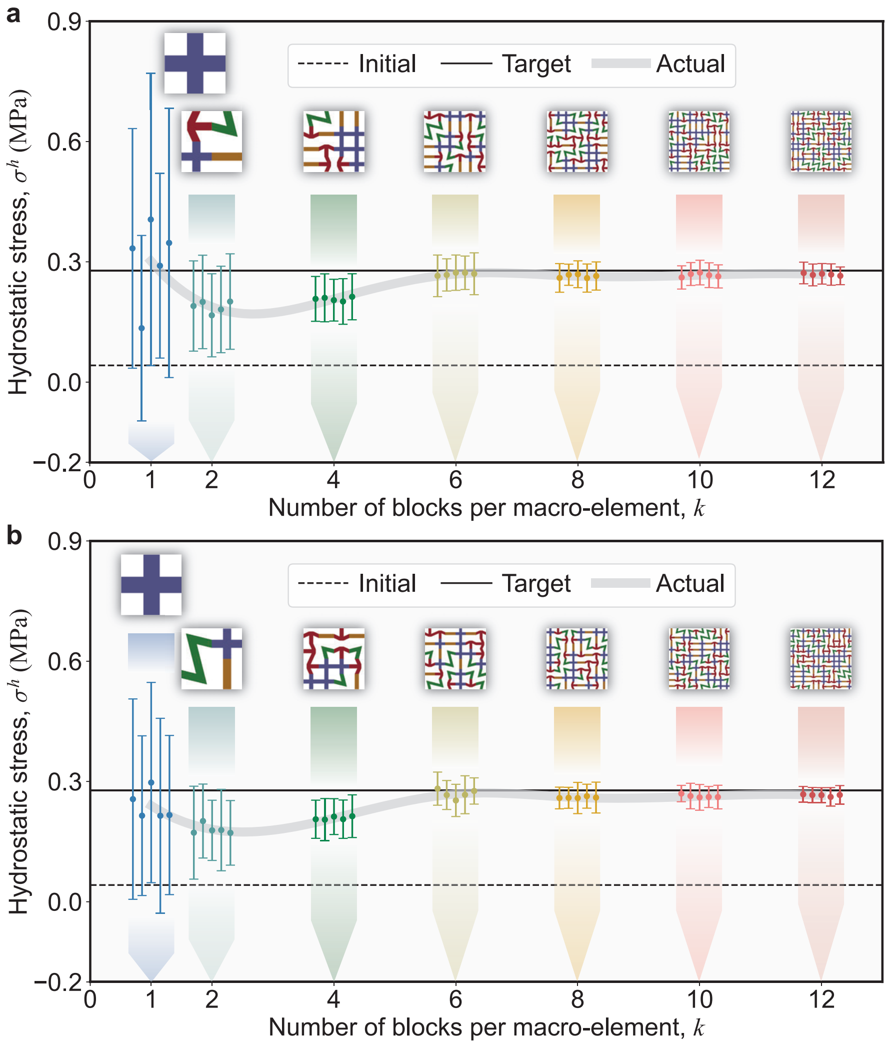

Supplementary Figure 10: **Stress convergence studies for mechanical stress modulation within distinct control regions.** **a**, Case 1; **b**, Case 2. The plots illustrate the relationship between the hydrostatic stress measure, denoted by σ^h (in MPa), and the number of basic building blocks in one direction within one microstructure. Each error bar represents one numerical sample. The circular dot of the error bar represents the mean hydrostatic stress in the control region, and the half length of the error bar represents the standard deviation.

Based on the average stress measures defined in (5)–(6), we perform the stress convergence studies for the generated irregular architected materials in Cases 1 and 2 in Figure 2. As depicted in Supplementary Figure 10, we examine the relationships between the hydrostatic stress, σ^h, and the number of basic building blocks in one direction within one microstructure, denoted by k (where k takes values 1, 2, 4, 6, 8, 10, and 12). The results indicate that the hydrostatic stress converges to the target value as k increases for both cases, which is consistent with the observation from the stress convergence study for Case 3 in the main manuscript.

Supplementary References

[1] K. Liu, R. Sun, C. Daraio, Growth rules for irregular architected materials with programmable properties, Science 377 (6609) (2022) 975–981. doi:10.1126/science.abn1459.

[2] A. Vigliotti, D. Pasini, Stiffness and strength of tridimensional periodic lattices, Computer Methods in Applied Mechanics and Engineering 229–232 (2012) 27–43. doi:10.1016/j.cma.2012.03.018.

[3] T. J. R. Hughes, The Finite Element Method: Linear Static and Dynamic Finite Element Analysis, Courier Corporation, 2012.

[4] D. P. Kingma, J. Ba, Adam: A Method for Stochastic Optimization (Jan. 2017). arXiv:arXiv:1412.6980, doi:10.48550/arXiv.1412.6980.

[5] Oliphant, E. Travis, Python for scientific computing, Computing in Science & Engineering 9 (3) (2007) 10–20.

[6] A. Paszke, S. Gross, F. Massa, A. Lerer, J. Bradbury, G. Chanan, T. Killeen, Z. Lin, N. Gimelshein, L. Antiga, et al., Pytorch: An imperative style, high-performance deep learning library, Advances in neural information processing systems 32.

[7] B. Bourdin, Filters in topology optimization, International Journal for Numerical Methods in Engineering 50 (9) (2001) 2143–2158. doi:10.1002/nme.116.

[8] M. P. Bendsoe, O. Sigmund, Topology Optimization: Theory, Methods, and Applications, Springer Science & Business Media, 2003.

[9] M. P. Bendsøe, O. Sigmund, Material interpolation schemes in topology optimization, Archive of Applied Mechanics 69 (9) (1999) 635–654. doi:10.1007/s004190050248.

[10] M. P. Bendsøe, N. Kikuchi, Generating optimal topologies in structural design using a homogenization method, Computer Methods in Applied Mechanics and Engineering 71 (2) (1988) 197–224. doi:10.1016/0045-7825(88)90086-2.

[11] A. Clausen, F. Wang, J. S. Jensen, O. Sigmund, J. A. Lewis, Topology Optimized Architectures with Programmable Poisson's Ratio over Large Deformations, Advanced Materials 27 (37) (2015) 5523–5527. doi:10.1002/adma.201502485.

[12] W. Li, F. Wang, O. Sigmund, X. S. Zhang, Digital synthesis of free-form multimaterial structures for realization of arbitrary programmed mechanical responses, Proceedings of the National Academy of Sciences 119 (10) (2022) e2120563119. doi:10.1073/pnas.2120563119.

[13] Z. Zhao, X. S. Zhang, Encoding reprogrammable properties into magneto-mechanical materials via topology optimization, npj Computational Materials 9 (1) (2023) 1–11. doi:10.1038/s41524-023-00980-2.

[14] W. Li, Y. Jia, F. Wang, O. Sigmund, X. S. Zhang, Programming and physical realization of extreme three-dimensional responses of metastructures under large deformations, International Journal of Engineering Science 191 (2023) 103881. doi:10.1016/j.ijengsci.2023.103881.

[15] M. W. Scroggs, J. S. Dokken, C. N. Richardson, G. N. Wells, Construction of Arbitrary Order Finite Element Degree-of-Freedom Maps on Polygonal and Polyhedral Cell Meshes, ACM Transactions on Mathematical Software 48 (2) (2022) 18:1–18:23. doi:10.1145/3524456.

[16] K. Svanberg, The method of moving asymptotes—a new method for structural optimization, International Journal for Numerical Methods in Engineering 24 (2) (1987) 359–373.

[17] H. Ebrahimi, M. Rabinovich, V. Vuleta, D. Zalcman, S. Shah, A. Dubov, K. Roy, F. S. Siddiqui, E. H. Schemitsch, H. Bougherara, R. Zdero, Biomechanical properties of an intact, injured, repaired, and healed femur: An experimental and computational study, Journal of the Mechanical Behavior of Biomedical Materials 16 (2012) 121–135. doi:10.1016/j.jmbbm.2012.09.005.

[18] C. B. Sullivan, A. A. Kaszynski, PyVista: 3D plotting and mesh analysis through a streamlined interface for the Visualization Toolkit (VTK), Journal of Open Source Software 4 (37) (2019) 1450. doi:10.21105/joss.01450.

[19] K. Jordahl, J. Van den Bossche, M. Fleischmann, J. Wasserman, J. McBride, J. Gerard, J. Tratner, M. Perry, A. Garcia Badaracco, C. Farmer, et al., geopandas/geopandas: v0. 8.1, Zenodo.

[20] S. Gillies, The shapely user manual, URL https://pypi. org/project/Shapely.

[21] B. Hassani, E. Hinton, A review of homogenization and topology optimization I—homogenization theory for media with periodic structure, Computers & Structures 69 (6) (1998) 707–717. doi:10.1016/S0045-7949(98)00131-X.
