# Supplementary material for: Modulate stress distribution with bio-inspired irregular architected materials towards optimal tissue support
Source: Nat Commun. 2024 May 21;15:4072. doi: 10.1038/s41467-024-47831-2 (PMC11109255; doi:10.1038/s41467-024-47831-2)
Supplement: Supplementary file 3 — Description of Additional Supplementary Files [file 41467_2024_47831_MOESM3_ESM.pdf]

Title: Supplementary Movie 1.
Description: Design optimization, virtual growth, and fabrication of optimized irregular architected materials with stress modulation for potential tissue support.

Title: Supplementary Movie 2.
Description: Lightweight architected materials for multifunctional stress modulation: design optimization, virtual growth, and comparison of numerical and experimental results.
